# Small Open Reading Frames: How Important Are They for Molecular Evolution?

**DOI:** 10.3389/fgene.2020.574737

**Published:** 2020-10-20

**Authors:** Diego Guerra-Almeida, Rodrigo Nunes-da-Fonseca

**Affiliations:** ^1^Institute of Biodiversity and Sustainability, Federal University of Rio de Janeiro, Rio de Janeiro, Brazil; ^2^National Institute of Science and Technology in Molecular Entomology, Rio de Janeiro, Brazil

**Keywords:** small ORF, junk DNA, coding potential maturation, causal roles, selected effects, non-coding RNA, alternative ORF, pervasive translation

## Introduction

Small Open Reading Frames (small ORFs/sORFs/smORFs) are important sources of putative peptides previously dismissed as being non-functional or junk DNA, as determined by early gene prediction methods. In fact, smORFs of <100 codons are possible coding sequences but sufficiently small to occur very frequently and randomly in genomes; thus, the detection of their coding potential and functional assessment is similar to a walk in the dark. Furthermore, while dozens of smORF peptides have been recently described as essential players in biological processes, many are reported to be potential non-functional products of junk DNA under pervasive translation, leading to the question: from what perspective is this lack of function assessed? In this context, it was recently suggested that non-functional smORF peptides might play a major role during *de novo* protein coding gene birth, but the evolutionary mechanism is still unclear. Thus, the role of pervasive translation of smORFs in molecular evolution remains puzzling. Here, we present interesting questions for debate and further investigation about the perspective of non-functional smORF peptides as underappreciated hotspots of molecular evolution in eukaryotes.

## Small Open Reading Frames: A Subtopic in the Discussion of Junk DNA Function

With respect to the evolution of molecular function, part of the DNA elements accumulate mutations by genetic drift; thus, the evolution of these elements is non-adaptive and neutral (Ohta, 2002). In some cases, the amount of neutrally evolving elements in junk DNA are analogous to the items on a menu available to natural selection (Knibbe et al., 2007; Faulkner and Carninci, 2009; Lynch et al., 2011). Interestingly, it was reported by the ENCODE consortium (the Encyclopedia of DNA Elements) that most of the human junk DNA exhibits some type of biochemical activity (ENCODE Project Consortium, 2012), but lacking adaptive relevance and selective pressure (Doolittle, 2013; Graur et al., 2013). Importantly, junk DNA represents 75–90% of the human genome (Graur, 2017).

Part of the junk DNA menu is composed of neutrally evolving smORF peptides. For instance, thousands of non-coding RNAs are generated by the extensive transcription coverage on junk DNA (ENCODE Project Consortium, 2007). Increasing evidence shows that thousands of smORFs undergo pervasive translation in transcripts annotated as non-coding or in untranslated regions (UTR) of mRNAs (e.g., Aspden et al., 2014; Ingolia et al., 2014). Interestingly, non-coding RNAs and ORFs lacking homologs were reported to be candidates for *de novo* evolution of protein coding genes (Tautz and Domazet-Lošo, 2011). Moreover, it was recently suggested that neutrally evolving smORF peptides might play a major role in this process (Ruiz-Orera et al., 2018), but the evolutionary mechanism remains to be determined (Ruiz-Orera et al., 2018; Singh and Wurtele, 2020). In this context, two previously proposed concepts used to discuss molecular function evolution are at the core of the junk DNA debate: “causal roles” and “selected effects” (Doolittle and Brunet, 2017), which will be discussed here in the context of smORFs and protein coding gene birth.

The “causal role” describes the activity performed by a neutrally evolving element by chance. For example, a hypothetical genomic sequence generated by a random nucleotide mutation to resemble a TATA box may be recognized and bound by transcription factors but does not trigger gene transcription (Griffiths, 2009; Graur et al., 2013). In other words, “causal roles” are non-adaptive phenotypes, their emergence is random, and they tend to rapidly disappear during evolution. On the other hand, “selected effects” describe the acquisition of adaptive phenotypes based on natural selection (Graur et al., 2013), such as canonical TATA boxes or ORFs that are translated into important proteins. In other words, “selected effects” are functionally relevant for cells.

Importantly, while natural selection drives adaptive evolution (selected effects), it is widely accepted that genetic drift drives junk DNA evolution, as well as the synonymous modifications in coding DNA sequences (CDS) and mutations in UTRs of mRNAs (Ridley, 2004).

## Discussion

Applying the aforementioned evidence and concepts, we discuss here a possible eukaryotic mechanism by which neutrally evolving smORFs advance proteome evolution and the evolutionary significance of smORFs.

**Figure 1:**
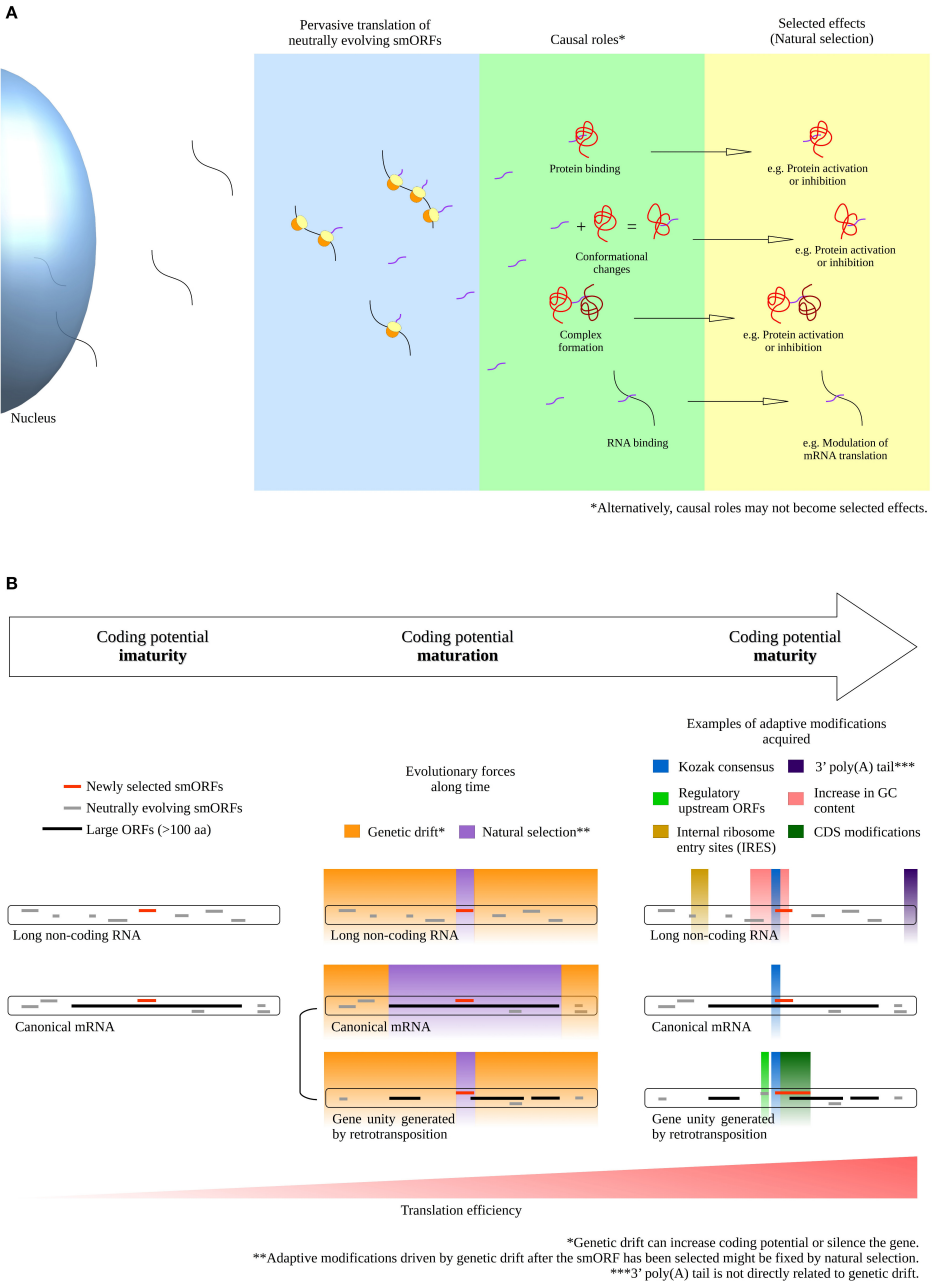
Phenotype selection and coding potential maturation of smORF transcripts. **(A)** Transition of smORF peptides from “causal roles” to “selected effects” after pervasive translation events. Pervasive translation of neutrally evolving smORFs possibly advances proteome evolution by exposing neutral phenotypes to natural selection under environmental pressure. **(B)** Scheme for coding potential maturation, a hypothetical mechanism that increase the translation efficiency of a mRNA after a smORF has been selected for (selected effect) in a transcript with suboptimal coding features. On the left, coding potential immaturity; in the middle, coding potential maturation; on the right, coding potential maturity. During the coding potential immaturity phase, newly selected smORFs are observed in transcripts with suboptimal coding features, either in long non-coding RNAs or as alternative smORFs in canonical mRNAs. Although canonical mRNAs exhibit optimal coding features, alternative smORFs are usually secondarily or pervasively translated; thus, some alternative smORFs may reside in suboptimal coding regions. During the coding potential maturation phase, natural selection and genetic drift may act in different parts of a transcript. While natural selection acts by fixing the selected parts, genetic drift acts by changing the non-coding parts of a transcript, as postulated by the nearly neutral theory (Ohta, 2002). Natural selection promotes fine-tuned adjustments to the selected phenotypes, such as synonymous mutations and CDS modifications. Genetic drift can establish adaptive mutations in a transcript by evolving sequences that potentially increase smORF translation, such as the Kozak consensus, regulatory upstream ORFs, internal ribosome entry sites (IRES) and increases in GC content. Additionally, other adaptive modifications not directly related to sequence mutations in transcripts might increase smORF expression, such as the 5′ cap, 3′ poly(A) tail, cis-regulatory elements in the genome and, in the case of alternative smORFs, independent gene unit generation by retrotransposition. Importantly, the acquisition of optimal coding features might be favored after the smORF has been selected for, because modifications driven by genetic drift could be fixed by natural selection if they improve the translation efficiency of the newly selected smORF. Before the smORF has been selected for, eventual optimal coding features acquired could rapidly disappear during genetic drift evolution without fixation. Alternatively, mutations evolved by genetic drift can silence the gene. Finally, smORFs reach the coding potential maturity phase when optimal coding features are acquired and translation efficiency increases. Consequently, the translation rate of smORF peptides is largely increased upon completion of the described process, contributing to the establishment of molecular innovations and protein coding gene birth.

Firstly, part of the roles performed by neutrally evolving smORF peptides possibly transit from “causal roles” to “selected effects” under environmental pressure, thereby exposing their neutral phenotypes to natural selection and triggering the evolution of new coding genes. Thus, when neutral smORF peptides are selected, they are no longer neutral (Ruiz-Orera et al., 2018). In other words, neutral smORF peptides may be special entrees on the junk DNA menu that are available for natural selection (Figure 1A).

Upon smORFs being selected for, they probably contain low adaptive relevance due to their non-coding transcript characteristics, such as low translation rate, lack of 3′-terminal processing and other suboptimal coding features (non-coding RNA features are reviewed in Quinn and Chang, 2016). This hypothesis is based on the fact that hundreds of smORFs are described as highly conserved but display low expression, low translation efficiency and are observed in transcripts with non-coding characteristics (Cabili et al., 2011; Aspden et al., 2014; Bazzini et al., 2014). However, the nearly neutral theory (Ohta, 2002) suggests that non-coding parts of fixed smORF transcripts are modified by random genetic drift, in some cases, producing small advantageous (or disadvantageous) adaptive effects throughout evolution; thus, we propose that, at a certain point, these modifications refine and elevate the coding potential of smORF transcripts and consequently enhance the adaptive relevance of their peptides, as seen in a large number of important smORF peptides recently discovered (e.g., Magny et al., 2013; Anderson et al., 2015; Lauressergues et al., 2015; Nelson et al., 2016; Pengpeng et al., 2017; Kim et al., 2018; Polycarpou-Schwarz et al., 2018; Chugunova et al., 2019; Tobias-Santos et al., 2019; Pang et al., 2020; Vassallo et al., 2020). Importantly, the acquisition of several optimal coding features might be favored after the smORF has been selected for, because modifications driven by genetic drift could be fixed by natural selection if they improve the translation efficiency of the newly selected smORF. Before the smORF has been selected for, eventual optimal coding features acquired in the nucleotide sequence could rapidly disappear during genetic drift evolution without fixation. Alternatively, nucleotide changes may negatively affect the coding potential and silence a gene. Optimal coding features include structural stabilization, emergence of Kozak consensus, internal ribosome entry sites (IRES), coverage by enhancers and, in some cases, the elongation of coding smORFs to enlarge the CDSs (coding DNA sequences) (Figure 1B). Recently, Couso and Patraquim (2017) proposed that at least a portion of functional smORFs are potential *de novo* precursors of large CDSs via a stop codon mutation pattern called “CDS elongation.”

Considering the supposition that the action of evolution is gradual, we propose that the aforementioned process be called “coding potential maturation” (Figure 1B). For example, smORF translation is widely reported in transcripts with long non-coding RNA (lncRNA) characteristics (Crappé et al., 2013; Ingolia et al., 2014; Ji et al., 2015; Mackowiak et al., 2015; Li et al., 2018; Lu et al., 2019). These lncRNAs exhibit smORF conservation in divergent species, hinting at natural selection fixation and indicating coding immaturity.

Another potential pathway of coding gene generation occurs via alternative smORFs in UTRs or overlapping the reference CDS of canonical mRNAs. In this scenario, alternative smORFs undergo pervasive translation or the act of translation itself is important for cis-regulatory purposes (Vanderperre et al., 2013; Wu et al., 2020). If the “causal roles” performed by neutrally evolving smORF peptides become “selected effects,” the alternative smORFs would generate independent gene units by retrotransposition, or they would be fixed as alternative smORFs in the original transcripts (Figure 1B). Hence, during retrotransposition events, at least a portion of the transcripts investigated on the basis of pseudogenization may, in fact, represent the maturation of new coding genes, as suggested by a report that pseudogenes can be translated into highly conserved smORF peptides (Ji et al., 2015).

smORFs might be sequence reservoirs potentially activated during the evolution of new phenotypic variations, especially during speciation. Importantly, speciation events have been associated with the evolution of new molecular phenotypes and new relationships with the environment (Bao et al., 2018). Thus, the amount of junk DNA and lncRNAs in cells deserves investigation not only as a random accumulation of sequences and translational noise but also as a repository of substrates to advance the evolution of new coding genes. Interestingly, polyploidization, or whole genome duplication (WGD) events, have been correlated with an increase in the adaptive potential of cells and organisms exposed to stressful conditions (Van De Peer et al., 2017). Unfortunately, thus far, studies of WGD have neglected the role and retention of smORFs during evolution, probably due to methodological difficulties in smORF identification.

However, the sequencing of several genomes based on comparative approaches has recently opened new avenues for smORF research. For instance, recent evolutionary studies performed by our group on the smORFs in the *mille-pattes*/*tarsalless*/*polished rice* (*mlpt*) gene, the most well-known smORF-containing gene in insects (Savard et al., 2006; Kondo et al., 2007; Pueyo and Couso, 2008, 2011; Cao et al., 2017; Ray et al., 2019), showed that a new ~80 amino acid smORF (smHemiptera) appeared during Hemiptera evolution (Tobias-Santos et al., 2019). Thus, this smORF in the polycistronic *mlpt* mRNA has been conserved for over 250 million years in the group, and it is not present in the genomes of other insect orders. We expect that new comparative analyses of genomes in the future will yield additional examples of order-specific smORFs, which might constitute an underappreciated reservoir of new genes and evolutionary innovations.

In summary, the study of smORFs has been considerably increasing during the last 5 years because of recent discoveries of important smORF peptides. Accordingly, the advent of ribosome profiling has allowed the discovery of many neutrally evolving and potentially non-functional smORFs undergoing pervasive translation, whose significance remains to be determined (Crappé et al., 2013; Aspden et al., 2014; Bazzini et al., 2014; Olexiouk et al., 2016). In this context, the intriguing question is posed: why would cells spend energy on transcription and translation of neutral and non-functional elements? There is probably more than one answer; however, considering the subjects discussed in this paper, we propose the following perspective: what if the pervasive translation of neutrally evolving smORF peptides composes an elegant mechanism to advance proteome evolution, especially during speciation events? If it does, then non-functional smORF peptides display an important function in an evolutionary sense. Based on this discussion, we suggest that the concept of functionality be revised in the context of smORFs.

## Author Contributions

DG-A and RN contributed equally to the writing of this manuscript. RN contributed to funding acquisition. All authors contributed to the article and approved the submitted version.

## Conflict of Interest

The authors declare that the research was conducted in the absence of any commercial or financial relationships that could be construed as a potential conflict of interest.
